# The impact of a school-based physical activity program on children's movement behaviors, aerobic fitness and motor competence: a follow up study

**DOI:** 10.3389/fspor.2025.1541862

**Published:** 2025-02-27

**Authors:** Moreno Bloch, Rita Cordovil, Luís P. Rodrigues, Clarice Martins, Maria L. Braga, Susana Vale, Rui Proença, João Brito, José Guilherme, Carlos Neto, André Seabra, Júlio A. Costa

**Affiliations:** ^1^ Faculdade de Motricidade Humana, Universidade de Lisboa, Lisboa, Portugal; ^2^ CIPER, Faculdade de Motricidade Humana, Universidade de Lisboa, Lisboa, Portugal; ^3^ Instituto Politécnico de Viana do Castelo, Escola Superior de Desporto e Lazer, Viana do Castelo, Portugal; ^4^ SPRINT Sport Physical Activity and Health Research & Innovation Center, Viana do Castelo, Portugal; ^5^ Faculty of Sports, Research Centre in Physical Activity, Health and Leisure, and Laboratory for Integrative and Translational Research in Population Health, University of Porto, Porto, Portugal; ^6^ Portugal Football School, Portuguese Football Federation, FPF, Oeiras, Portugal; ^7^ Politécnico do Porto—Escola Superior de Educação, Porto, Portugal; ^8^ School D. Carlos I, Sintra, Portugal; ^9^ Centre of Research, Education, Innovation and Intervention in Sport, Faculty of Sport, University of Porto, Porto, Portugal

**Keywords:** sedentarism, childhood, exercise, physical activity program, health

## Abstract

**Objective:**

This study evaluated the one-year impact of a 12-week school-based physical activity (PA) intervention, which added one extra hour of structured PA per week, on children's movement behaviors, aerobic fitness, and motor competence (MC).

**Methods:**

A longitudinal comparison between an experimental group (EG) and a control group (CG) was conducted at baseline (PRE), mid-intervention (DUR), post-intervention (POST), and one-year follow-up (FUP). The study was conducted in a single school without randomization. Movement behaviors (accelerometry), aerobic fitness (YYIR1C), and MC (MCA battery) were assessed. Due to school changes and parental noncompliance, the sample decreased from 38 to 23 participants (EG; *n* = 13; CG; *n* = 10), a 39% dropout rate.

**Results:**

One year post-intervention, the EG spent more time in moderate-to-vigorous PA (MVPA; + 41 min/day), less time in SED (−234 min/day), slept longer (+103 min/night), covered greater distances in the YYIR1C (+174 m), and achieved higher MC scores (+19% on the MCA battery test) compared to the CG. Although no significant differences in body weight were observed, the EG showed improved body mass index (BMI; −5 kg/m^2^) and a lower body fat percentage (−10%) when compared to the CG.

**Conclusion:**

The findings indicate that the benefits of a 3-month school-based PA intervention, which included an additional hour of physical education per week, can have sustained benefits for movement behaviors, physical fitness, and MC in children one year post-intervention. However, the small sample size and lack of randomization limit the generalizability of the results. Future research should employ larger, randomized trials to better assess the long-term impact of PA interventions on children’s health and fitness outcomes.

## 1 Introduction

Childhood physical activity (PA) levels are declining globally (1). The World Health Organization (WHO) recommends that children and adolescents achieve at least 60 min of moderate to vigorous PA daily (2). In Portugal, only 16% of the girls and 27% of the boys aged 11 meet the recommended guidelines for physical activity, as reported in the WHO growth reference standards (3). Additionally, in the same age group, 26% of girls and 36% of boys are classified as overweight or obese based on these standards (3). Childhood and adolescent movement behaviors are crucial determinants of lifelong health (4). Sedentary behavior (SED) is defined as activities with energy expenditure lower or equal to 1.5 metabolic equivalents (MET) while sitting or lying down during the waking period (5). Prolonged SED during childhood and adolescence is as a predictor of SED in adulthood and is directly related to the development of chronic diseases such as diabetes, hypertension, overweight or obesity and metabolic syndrome (6).

Given the substantial amount of time children and adolescents spend in schools, this environment presents an ideal setting to promote health-enhancing movement behaviors for nearly all school-aged individuals (7). School-based interventions frequently employ multicomponent approaches that not only focus on improving PA levels but also target related health parameters, such as sleep, aerobic fitness, and motor competence (MC) (8). These holistic strategies acknowledge the interconnection of these health aspects and their potential to mutually influence one another (9).

Frequent engagement in PA is also important to promote good sleep duration and quality. Inadequate sleep patterns have been associated with reduced PA levels in children (10), while increased PA has been related with improved sleep duration and quality, establishing a reciprocal relationship between these two important health behaviors (11). Also, reduced sleep has been associated with a heightened risk of obesity in children. Studies indicate that children who sleep less than eight hours per night face nearly three times the risk of obesity compared to those who achieve adequate sleep (12).

Moreover, childhood is a critical time for the development of MC, which not only enables children and adolescents to participate successfully in various physical tasks, games, and sports, but also significantly influences their emotional, affective, social, and academic development (13). This foundational skill set supports their ability to engage meaningfully with peers, build self-confidence, and achieve greater academic performance, highlighting its multidimensional importance to impacts children's physical, social, and cognitive development (14).

Concerning cardiorespiratory fitness (CRF), more active children tend to have better CRF, and children with better CRF are more likely to engage in PA (13). Adequate CRF levels during youth and their improvement have been associated with a lower risk of developing obesity and cardiometabolic disease later in life (15). Results suggest that early intervention and supportive prevention strategies (e.g., in schools) that promote CRF can help children maintain or achieve a healthy status, thereby reducing future health problems (16). Therefore, the aim of this study is to evaluate the long-term effects of a school-based PA intervention program on primary school children. Specifically, the study investigates how the program influenced movement behaviors, aerobic fitness, and MC one year after (i.e., follow-up) its conclusion.

## 2 Materials and methods

### 2.1 Study design

One year after completing the main phase of the Super Quinas program (17)—a 12-week intervention designed to improve daily movement behaviors, aerobic fitness, and motor competence (MC) in primary school children—we returned to the same school to reassess these outcomes. This Follow-Up Phase (FUP) concluded in March 2024, aiming to evaluate the long-term sustainability of the program's effects without additional intervention.

The main phase of the study (baseline to 12-week follow-up) involved three data collection points: PRE (January 2023, 1st week), DUR (February 2023, 6th week), and POST (March 2023, 12th week). Throughout these phases, weekly movement behaviors, aerobic fitness, and MC were assessed. A detailed description of the methodology and study design can be found in (17).

This pilot project was conducted in 44 schools across Portugal, involving over 1600 children aged 6–10. All participants completed MC assessments during both the PRE and POST phases. Additionally, a subgroup of 38 children underwent more specific evaluations of movement behaviors, aerobic fitness, and MC.

One year later, during the FUP, 23 children from this subgroup were reassessed using the same measures. To ensure consistency, 4th grade students were selected based on parental interest and availability. Both experimental (EG) and control (CG) groups were randomly chosen by the school director.

### 2.2 Participants

Participants were selected from a primary school in Sintra, Portugal, chosen for its accessibility and feasibility for data collection. The study focused on 4th grade students who had previously taken part in the 12-week school-based physical activity (PA) intervention program (Super Quinas) (17).

During the main phase (baseline to 12-week follow-up), the EG consisted of 19 children (8 girls, 11 boys), while the CG included 19 children (9 girls, 10 boys). Rather than random assignment, participants were allocated to the EG and CG based on their existing class groupings within the same grade. The EG received an additional 60 min of structured PA per week, whereas the CG followed the standard school curriculum. This class-based grouping was determined by logistical and structural constraints within the school.

One year after the intervention, during the Follow-Up Phase (FUP), 23 children remained in the study: 13 in the EG (7 boys, 6 girls) and 10 in the CG (4 boys, 6 girls), with a mean age of 10.8 years. This represented a 39% dropout rate compared to the main phase (17). The primary reasons for dropout were school transfers (*n* = 12; 80%) and parental noncompliance (*n* = 3; 20%).

To be eligible, students had to be enrolled in the selected school during both the intervention and follow-up phases. Exclusion criteria included taking medication or having a clinical condition that contraindicated PA. However, no children were excluded based on these criteria.

Before data collection, participants and their parents/legal guardians were fully informed about the study's objectives, duration, intervention type, and potential risks and benefits. Parents were reassured that any injuries or unexpected issues would be promptly addressed, with guidance provided for medical consultation if necessary. They were also informed of their right to withdraw their child from the study at any time without consequences. Written informed consent was obtained from legal guardians, and verbal assent was collected from the children. The study was approved by the Ethical Committee of the Portugal Football School, Portuguese Football Federation (protocol number CEPFS 17.2022).

### 2.3 Intervention content

#### 2.3.1 Baseline to 12-week follow-up

The 12-week intervention program aimed to improve primary school children's movement behaviors, aerobic fitness, and MC. The EG participated in the Super Quinas intervention, totaling 120 min of PA per week—including their regular 60 min physical education class plus an additional 60 min structured PA session (17). In contrast, the CG followed their standard schedule, which included 60 min of physical education and 60 min of non-PA extracurricular activities (e.g., languages, art, or other non-PA subjects) (17).

During the intervention, the additional 60-minute session for the EG was structured as follows:

• Warm-up and Individual Skill Development (20 min):
  - Focused on fundamental movement skills, including running, jumping, balancing, crawling, and climbing to enhance body awareness.
  - Included ball manipulation drills, such as dribbling, controlling, throwing, and kicking at both fixed and moving targets.
• Partner and Small Group Activities (30 min):
  - Involved collaborative exercises in pairs or small groups to reinforce body awareness and ball-handling skills.
  - Featured cooperative games that encouraged skill development through teamwork.
• Group Games (10 min):
  - Integrated pre-sport and small-sided games, emphasizing passing, receiving, dribbling, shooting, offensive and defensive strategies.
  - Promoted teamwork and tactical understanding through interactive play.

This structured approach provided a progressive learning experience, combining individual skill-building with cooperative and game-based activities.

#### 2.3.2 Post-intervention to follow-up phase

Following the conclusion of the intervention in March 2023, participants advanced to 5th grade in September 2023. By the Follow-Up Phase (FUP) in March 2024, both the EG and CG followed the same physical education schedule, consisting of two 60 min PE classes per week (a total of 120 min per week).

No additional structured intervention was introduced during the one-year follow-up period, allowing researchers to evaluate the long-term effects of the original Super Quinas program under standard school conditions.

### 2.4 Measures

#### 2.4.1 Body composition

Body composition was assessed using an InBody 270 bio-impedance scale with an 8 Electrode Tetrapolar Electrode System with frequencies of 20 and 100 kHz. This allowed for the measurement of weight, body fat mass (%) and body mass index (BMI) (18). A portable stadiometer (Seca 213, Germany) was used to measure height. All measurements were conducted with participants lightly dressed (underwear and a t-shirt) and barefoot.

#### 2.4.2 Movement behaviors

Daily movement behaviors were estimated using a tri-axial accelerometer (ActiGraph, model GT3X, Acticorp Co., Pensacola, FL, USA) during four time points: baseline (i.e., PRE); middle of the intervention program (i.e., DUR); at the end of the main phase (i.e., POST); and one year after (i.e., FUP). Participants wore the accelerometer for 7 consecutive days (i.e., Monday to Sunday) at each time point, secured with an elastic belt around the waist. Children were instructed to wear the device continuously for 24 h, removing it only during bathing, water-based activities, or high-risk sports (e.g., martial arts). For data analysis, a minimum of 4 valid days were considered (3 weekdays and 1 weekend day). Valid records needed a minimum of 8 h of recording per day (19, 20). Wear time validation was calculated using Troiano defaults (21, 22).

PA intensity was classified based on the Evenson Children cut-points (19): *light* PA (101 to ≥2,295 counts per min), *moderate* PA (≥ 2,296 counts per min), and *vigorous* PA (≥ 4,012 counts per min) (20, 23).

Sleep monitoring was also assessed with the accelerometers, worn on the non-dominant wrist during the night sleep (24). Sleep variables were recorded nightly over 7 consecutive days for each assessment point (i.e., PRE, DUR, POST and FUP). Data was analyzed using the Sadeh's algorithm (25, 26). Sleep indices included sleep duration (amount of sleep in hours) and sleep efficiency (percentage of time in bed spent asleep) (25). According to the National Sleep Foundation (27, 28), a sleep duration < 8 h was considered insufficient, and a sleep efficiency ≤ 65% was considered an poor sleep quality for children (27, 28).

#### 2.4.3 Aerobic fitness

Aerobic fitness was assessed using the Childreńs Yo-Yo test (YYIR1C) at PRE (1st week), DUR (6th week), POST (12th week) and FUP (one year after), for the EG and CG. The YYIR1C uses a 16-meter shuttle run (instead of 20 meters) and a 4-meter walk (instead of 5 meters) during the 10-second active recovery period (29, 30). It uses the same acoustic progression, but shorter distance compared to Yo-Yo intermittent recovery test in its level 1 (YYIR1) version (30, 31). This test was developed for accounting for differences in running economy in children and has recently been reported to be a reliable and valid (construct validity) test for children's of either sex (31). Testing was conducted in the school gym at the same time of day, to control for circadian variation. All children were acquainted with the assessment procedures during physical education classes the week before testing.

#### 2.4.4 Motor competence

Motor competence was assessed using the MCA battery (32, 33), which comprises six tests, two for each component of motor competence: stability (lateral jumps and shifting platforms), locomotor (standing long jump and 10 m shuttle run), and manipulative (ball kicking velocity and ball throwing velocity). All tests are quantitative (product oriented), without a marked developmental (age) ceiling effect, and based on the child's feasible execution of motor tasks.

Testing was conducted in small groups (about five children per task), after a 10-minute warm-up (33, 34). Examiners were previously trained in administering all tests, and the following requirements were standardized: (a) a proficient demonstration of each test technique was provided along with a verbal explanation; (b) every participant tried each task before the actual test administration; (c) the instructions emphasized that children should try to perform the task at their maximum capacity (e.g., “as fast as possible” for the stability tests and 4 × 10 shuttle run; “as far as possible” for the standing long jump; and “as hard as possible” for the manipulative tests); and (d) motivational, but no verbal feedback was provided (33, 34).

Results on each test were transformed into percentile scores by age and sex, according to the MCA norms (33). Subscales scores were calculated by averaging the percentile values of the two constituent tests, and the Total MCA score was calculated by averaging the subscales’ scores. This means that all MCA scores (tests, subscales, and total MCA) represent percentile positions according to age and sex.

### 2.5 Statistical analyses

Sample distribution was tested using the Shapiro–Wilk test for movement behaviors, aerobic fitness, body composition and motor competence, for the EG and CG, at PRE, DUR, POST and FUP. A linear mixed model analysis was performed to examine differences in *moderate* to *vigorous* PA (MVPA), sleep duration and efficiency indices, total distance covered in the aerobic fitness test and the total MC, between the EG vs. CG, at PRE, DUR, POST and FUP. A α-level of 0.05 was set as significant for all statistical comparisons. The PRE, DUR, POST and FUP moments were included as a fixed effect and player identity (subject ID) as the random effect, between EG vs. CG. Furthermore, among the recommended variance-covariance structure models, compound symmetry was selected according to the smallest Akaike Information Criterion assessment (35) based on the Maximum Likelihood method. Pairwise comparisons (Bonferroni) were used to show the mean differences for MVPA, sleep duration and efficiency, total distance performed in the aerobic fitness test and the total MC between EG vs. CG.

## 3 Results

Table 1 presents the anthropometric characteristics of the participants. At the FUP moment, the EG showed improved body mass index (body max index; −5 kg/m^2^) and a lower body fat percentage (−10%) compared to the CG although no significant differences were observed in body weight.

**Table 1:** Characteristics of participants in the EG and CG, including age, weight, height, body fat mass and body mass index (BMI).

	PRE (1st week) EG (*n* = 19)	PRE (1st week) CG (*n* = 19)	POST (12th week) EG (*n* = 19)	POST (12th week) CG (*n* = 19)	FUP (one year later) EG (*n* = 13)	FUP (one year later) CG (*n* = 11)
Age (years)	9.1 (9–10)	9.1 (9–10)	9.2 (9–10)	9.2 (9–10)	10.8 (9–11)	10.8 (9–11)
Weight (kg)	33.1 (24.8–43.9)	33.2 (24.7–44.1)	27.3 (20.6–41.7)^a,b^	36.9 (25–44.9)^a^	36.2 (26.1–53.6)^a^	38.9 (29.6–55.9)^a^
Height (m)	143 (132–156)	144 (134–158)	146 (138–158)	147 (136–159)	149 (141–166)^a^	148 (143–164)^a^
Body fat mass (%)	22 (12–41)	23 (12–39)	14 (11–21)^a,b^	26 (12–44)^a^	17 (14–24)^a,c,d^	27 (10–33)^a^
BMI (kg/m^2^)	17.5 (15.1–24.3)	18.1 (13.4–22.6)	14.8 (11.3–20.7)^a,b^	20.5 (16.8–24.7)^a^	16.1 (15.2–25.5)^a,c,d^	20.1 (18.8–25.6)^a^

Values are expressed in mean 95% confidence interval. EG, experimental group; CG, control group; BMI, body max index.

^a^ Significant differences compared to PRE.

^b^ Significant differences compared to CG in POST.

^c^ Significantly different compared to EG in POST.

^d^ Significantly different compared to FOLLOW-UP CG.

**Figure 1:**
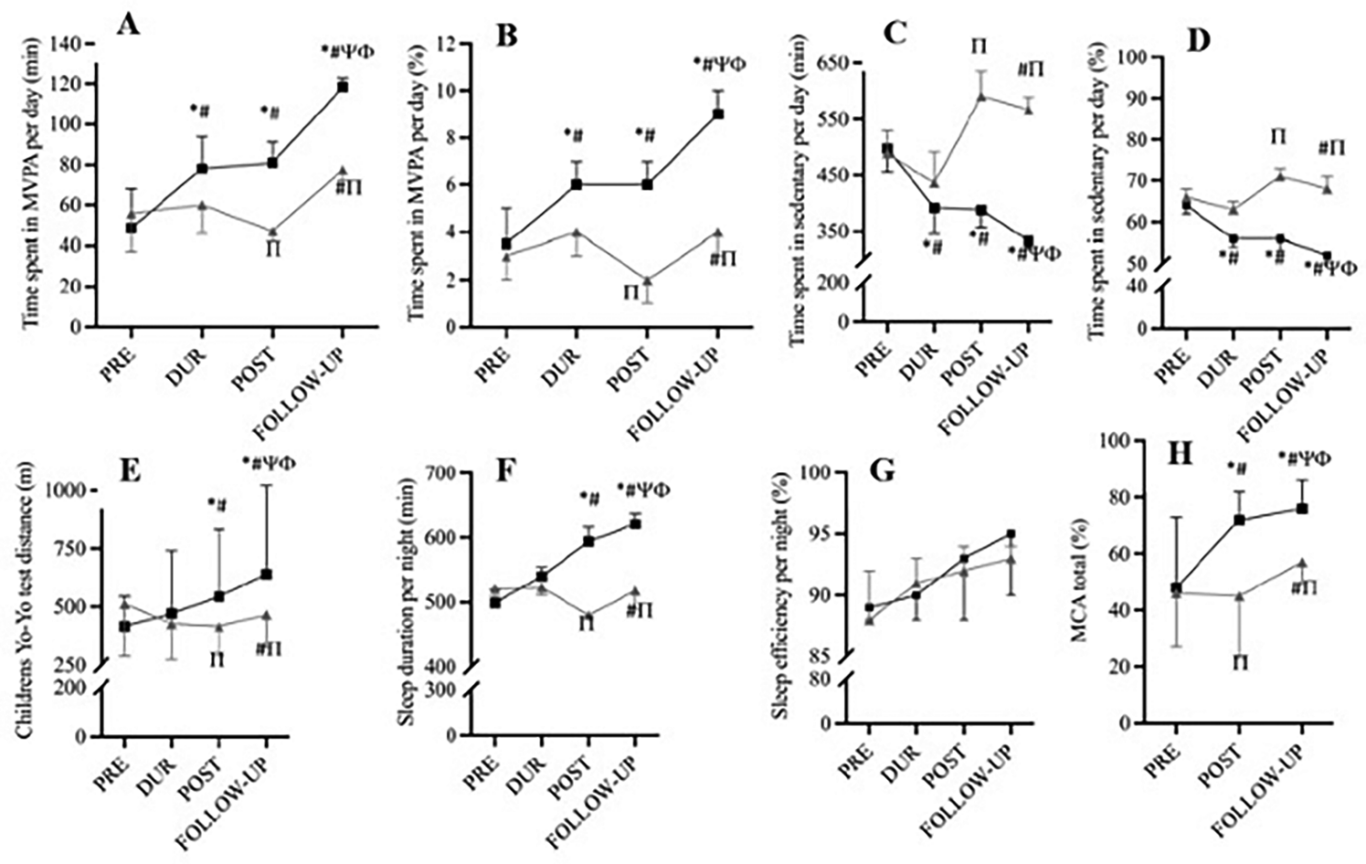
Changes in physical activity **(A,B)**, sedentary behavior **(C,D)**, aerobic fitness **(E)**, sleep **(F,G)**, and motor competence **(H)** over time in the experimental group (black lines; *n* = 13) and control group (gray lines; *n* = 10). Statistical significance (with 95% confidence interval) is indicated by symbols (*, #, *Ψ*, *Π*, *Φ*), with details provided in the figure legend.

From POST to FUP the CG showed the following changes:: *moderate* to *vigorous* physical activity/day—increased from an average of 47 to 77 min (*p* < 0.001); sedentary behaviour—slightly reduced the average time from 591 to 567 min/day (*p* < 0.001); Sleep duration—increased from an average of 480 to 518 min (*p* < 0.001); Sleep efficiency increased from 92% to 93% (*p* > 0.05); Aerobic fitness (YYIR1C test)—improved from an average of 416 meters to 466 meters (*p* < 0.001); MC (MCA battery)—increased from the 45th to 57th percentile (Figure 1).

In the same period, EG showed the following improvements: *moderate* to *vigorous* physical activity/day—increased from an average of 81 to 118 min (*p* < 0.001); sedentary behaviour—reduced from an average of 388 to 333 min/day (*p* < 0.001); Sleep duration—increased from an average of 594 to 621 min/night (*p* < 0.001); Sleep efficiency—increased from 93% to 95% (*p* > 0.05); Aerobic fitness (YYIR1C test)—increased from an average of 544 meters to 640 meters (*p* < 0.001); MC (MCA battery)—increased from the 72th to the 76th percentile (*p* < 0.001) (Figure 1).

## 4 Discussion

The aim of this research was to evaluate the impact of a school-based PA intervention program on primary school children's movement behaviors, aerobic fitness, and MC one year after its conclusion. The intervention targeting the EG consisted of 12 weeks between January and March of 2023.

During this period the CG maintained their regular schedule, which included one 60-min PE class, and 60-min extra-curricular activity class per week (i.e., languages, art, or other non-PA activities). From September 2023, both groups began their 5th school year, thus having two PE classes of 60 min per week.

In the POST moment there were significant positive effects on several parameters when comparing the CG to the EG. The EG demonstrated significant improvements in body fat mass, and body mass index compared to the CG. Additionally, the EG exhibited positive changes in movement behaviors, and aerobic fitness, bringing them closer to or within the WHO's guidelines for children in their age group (2).

This aligns with the findings of other school-based PA interventions lasting 11 (36, 37) and 12 weeks (38), highlighting the importance of movement behavior interventions in improving overall child health and well-being. These studies demonstrated positive effects on body composition, further supporting the value of such interventions. Finally, we now address the impact of this PA school program, one year after its completion.

### 4.1 Motor competence

Stodden et al. propose a theory called spiral of engagement, that can go in a positive or negative direction. It states that children with low MC tend to perceive themselves as less apt to engage in PA and, therefore, do it less frequently. Resulting in high levels of physical inactivity that may place these individuals at risk for being obese during later childhood, adolescence, and adulthood. Conversely, children with better motor skills are more likely to engage in PA frequently, developing better MC, leading to better overall health (39).

Our results seem to concur with this proposition, since the EG progressed from the 48th percentile in total MCA at baseline (PRE), to 72nd percentile at post-intervention (POST), and then to the 76th percentile after one year at FUP. The CG started at the 46th percentile at PRE, and was at the 45th percentile at POST, period in which they were not participating in the intervention. One year after the ’Super Quinas’ program—when they had two hours of physical education classes per week for the last 6 months—the CG's average score on total MCA improved to the 57th percentile.

The sustained improvement in the EG after one year is noteworthy. It suggests that the intervention may have effectively supported the development of MC in the EG. Additionally, this long-term improvement underscores the importance of continued PA to maintain and further enhance motor skills, suggesting that interventions with a lasting impact, could contribute to long-term health and skill development (40).

The comparison between the CG and EG highlights the effectiveness of the intervention in fostering improvements in MC. The EG's significant progress (from the 48th to the 76th percentile) far exceeds the modest improvements seen in the CG (from the 45th to the 57th percentile). This suggests that the targeted intervention, which involved more hours of structured PA and focused skill development, was a key factor in the improvements observed in the EG which concurs with (41), that affirms guided school-based PA programs can be influential in promoting physical fitness and MC among early adolescent students (41).

### 4.2 Movement behaviors

Similarly, PA also manifests an interdependence with cardiorespiratory fitness and sleep. Where physically active individuals tend to sleep more and better and, people with better sleep habits have more disposition for the practice of PA (42). Short sleep duration has consistently been shown to be associated with obesity among children and youth (43), it may influence the development of obesity through several possible biological pathways, including increased energy intake and/or decreased energy expenditure, due to SED (44). Moreover, children with lower levels of aerobic fitness tend to spend more time in SED and have less hours of sleep per night (10). Promoting regular PA can be an effective approach to improving sleep health and overall well-being (45).

According to our findings., the time spent in moderate to vigorous PA, increased for both the CG and EG across the study period (i.e., FUP). More specifically, at FUP, the CG reached 77 min, and the EG reached 118 min (41 min more), respectively, compared to the first evaluation, i.e., PRE moment. In intragroup comparison, the CG showed a 39% increase in time spent in MVPA from PRE to FUP, while the EG showed a 57% increase during the same period. Concerning the time spent in SED, the CG averaged 489 min on PRE, while EG averaged 497 min. On FUP, both groups reduced the sedentary time, but the difference remained substantial: the CG averaged 567 min, while the EG averaged 333 min.

These findings are consistent with the framework suggested by (46), wherein higher PA levels combined with lower SED are associated with healthier profiles. By reducing sedentary time and simultaneously increasing PA, the EG in the current study demonstrated a behavioral shift that is directly aligned with these cardiometabolic health recommendations, underscoring the potential of targeted interventions to promote long-term health in school-aged children (46).

Regarding sleep, on FUP, the CG had an average of 518 min of sleep per night and 93% of sleep efficiency compared to the first evaluation, i.e., PRE moment. Although the variation in average sleep duration was not significant from PRE to FUP, the CG showed a 5% improvement in sleep efficiency. The EG at FUP increased to 621 min of sleep duration and 95% of sleep efficiency. From PRE to POST, the EG showed an increase of 24% in sleep duration and 6% in sleep efficiency, compared to the first evaluation, i.e., PRE moment.

Overall, these findings on improved sleep duration and efficiency, especially in the EG, even after one year, align with research by (45), which highlights the beneficial role of moderate-intensity PA in managing sleep.

### 4.3 Aerobic fitness

Regarding aerobic fitness, assessed through Yo-Yo test (YYIR1C), the CG had an average of 512 m on PRE, 416 m on POST and 466 m on FUP. The EG had an average of 416 m on PRE, 544 m on POST and 640 m on FUP. From POST to FUP the CG and EG improved the aerobic fitness by 12% and 18%, respectively. These finds could be due to an increase in PE time, since the children's now from the 5th grade, began having two physical education classes per week, of 60 min each. Similar results were reported by (47), who demonstrated that a PA program with follow-up in children improved both cardiovascular risk factors and PA levels. Furthermore, the sustained improvements observed in the EG may be attributed to several mechanisms. The intervention's focus on diverse motor tasks likely enhanced motor skill development, creating a positive feedback loop of increased PA and competence, as suggested by Stodden et al. spiral of engagement theory. Additionally, the structured physical activities and supportive school environment may have facilitated habit formation and social reinforcement, fostering long-term adherence to active behaviors and improved aerobic fitness (47).

## 5 Strengths and limitations

It is important to acknowledge both the strengths and limitations of this study. This research contributes to the existing knowledge by exploring the potential of a school-based intervention to improve movement behaviors, aerobic fitness, and MC among primary school children. Moreover, the use of objective instruments (e.g., accelerometers), further strengthens the design of our study, which enhance the reliability and credibility of the findings. Accelerometry provided precise data on movement behaviors, while the Children's Yo-Yo Test and MCA battery objectively assessed aerobic fitness and MC, respectively. These tools reduced potential biases associated with self-reported data, ensuring robust and reproducible results. Additionally, the longitudinal design of this study offers significant value by providing critical insights into the sustainability of intervention effects over time. Tracking outcomes one-year post-intervention allowed for a deeper understanding of the long-term benefits of the program, highlighting its potential to create lasting improvements in PA, fitness, and MC.

However, there are limitations to consider. The small sample size and lack of randomization increase the risk of bias and limit the ability to draw broader conclusions. Additionally, the absence of data on socioeconomic status, extracurricular activities, and family dynamics restricts the understanding of contextual factors that may influence outcomes. Furthermore, while data on sex distribution were collected, the analysis did not include a stratified approach by sex regarding the small sample size. Finally, part of the results may be consequence of the natural process of biological maturation.

Therefore, future studies should include larger, more representative samples, employ randomized designs, and collect comprehensive data on contextual variables to better explore the interplay between school-based interventions and external influences on children's health and fitness outcomes.

## 6 Conclusion

The findings indicate that the benefits of a 3-month school-based PA intervention, which included an additional hour of physical education per week, can have sustained benefits for movement behaviors, physical fitness, and motor competence in children one year post-intervention. However, the small sample size and lack of randomization limit the generalizability of the results. Future research should employ larger, randomized trials to better assess the long-term impact of PA interventions on children's health and fitness outcomes.

## Data Availability

The raw data supporting the conclusions of this article will be made available by the authors, without undue reservation.
